# Hypertension With Brachydactyly Syndrome: A Case Report

**DOI:** 10.7759/cureus.8329

**Published:** 2020-05-28

**Authors:** Rizwan Asim, Anand Reddy, Olga Grigorieva Olson, Joshua A Ronen, Vivek Prasad

**Affiliations:** 1 Internal Medicine, Texas Tech University Health Sciences Center at Permian Basin, Odessa, USA; 2 Nephrology, Medical Center Health System, Odessa, USA; 3 Internal Medicine, Permian Internal Medicine Associates, Odessa, USA

**Keywords:** stroke, genetic screening, glomerulosclerosis, neurovascular anomaly, salt-independent hypertension, bilginturan syndrome, htnb, hypertension with brachydactyly

## Abstract

We describe the case of a 23-year-old African American male who presented to the emergency department complaining of unremitting dyspepsia for the last four months. His blood pressure was incidentally found to be 230/157 mm Hg. The initial admitting diagnosis in the intensive care unit was hypertensive “emergency” as he had also displayed acute kidney injury that was deemed to be superimposed on chronic kidney disease. While the diagnostic work-up of his hypertension was inconclusive, physical examination was impressive for the presence of brachydactyly of the bilateral hands, especially the fourth digits. His feet appeared grossly normal. X-rays (XRs) of the bilateral hands revealed absent distal phalanges and fused middle and distal phalanges of the second digits. XRs of the bilateral feet showed similar findings in addition to the absence or hypoplasia of the lateral cuneiform bones. His family medical history was unknown as the patient was adopted and did not have contact with his biological parents. Given these findings in the setting of uncontrolled hypertension in a young adult, he was diagnosed with hypertension with brachydactyly syndrome.

## Introduction

Hypertension with brachydactyly (HTNB) or Bilginturan syndrome is a rare autosomal dominant condition with multiethnic predominance that results in age-dependent and salt-independent hypertension [1]. If left untreated, it results in recurrent strokes in patients less than 50 years of age and a subsequent high mortality rate. Cases of HTNB have been described in North American and Middle Eastern families (i.e., Turkish) [1]. Such patients, if regularly followed by a primary care provider, can be diagnosed and treated during childhood and adolescence. Genetic testing for this syndrome in patients with a supportive phenotype can facilitate family screening and pre-pregnancy genetic counseling.

## Case presentation

A 23-year-old African American male with a medical history of chronic migraines presented to the emergency department complaining of unremitting dyspepsia for the last four months. He also complained of fatigue, ataxia, and orthopnea. He denied diplopia, chest pain, exertional dyspnea, palpitations, lightheadedness, nausea, vomiting, hematemesis, or hematochezia. The patient denied smoking, alcohol, illicit drug use, non-steroidal anti-inflammatory drug (NSAID) use, and recent intravenous (IV) contrast exposure. His family medical history was unknown as the patient was adopted and had no contact with his biological parents. His blood pressure was incidentally found to be 230/157 mm Hg. The patient was admitted to the intensive care unit with the initial diagnosis of hypertensive emergency for which nicardipine drip was instituted. Lab results also showed acute kidney injury (AKI) that was deemed to be superimposed on chronic kidney disease (CKD), among other findings (Table 1).

**Table 1:** Laboratory results on admission to the intensive care unit Note: values are normal unless otherwise indicated. WBC, white blood cells; L, low; MCV, mean corpuscular volume; RDW, red blood cell distribution width; MCHC, mean corpuscular hemoglobin concentration; H, high; TSH, thyroid-stimulating hormone; NT-proBNP, N-terminal pro-B-type natriuretic peptide.

Test	Results	
WBC	10.1 x 10^3^/mL	
Hemoglobin	13.3 g/dL (L)	
Hematocrit	39.2% (L)	
Platelets	268 x 10^3^/uL	
MCV	82.2 fL	
RDW	14.2%	
MCHC	33.9 g/dL	
Glucose	90 mg/dL	
Blood urea nitrogen	21 mg/dL	
Creatinine	1.8 mg/dL (H)	
Creatinine clearance	74 cc/min (L)	
Sodium (mmol/L)	134 mEq/L (L)	
Potassium (mmol/L)	2.9 mEq/L (L)	
Chloride (mmol/L)	98 mEq/L	
Cortisol	27.3 mcg/dL (H)	
TSH	1.51 uIU/mL	
NT-proBNP	5,510 pg/mL (H)	
Urine drug screen	Negative	
Urinalysis	20 ketones, 50 glucose, > 500 protein	
Urine specific gravity	1.1015	
Troponin (ng/mL)	0.16 ng/mL -> 0.12 ng/mL -> 0.35 ng/mL (H)	

**Figure 1:**
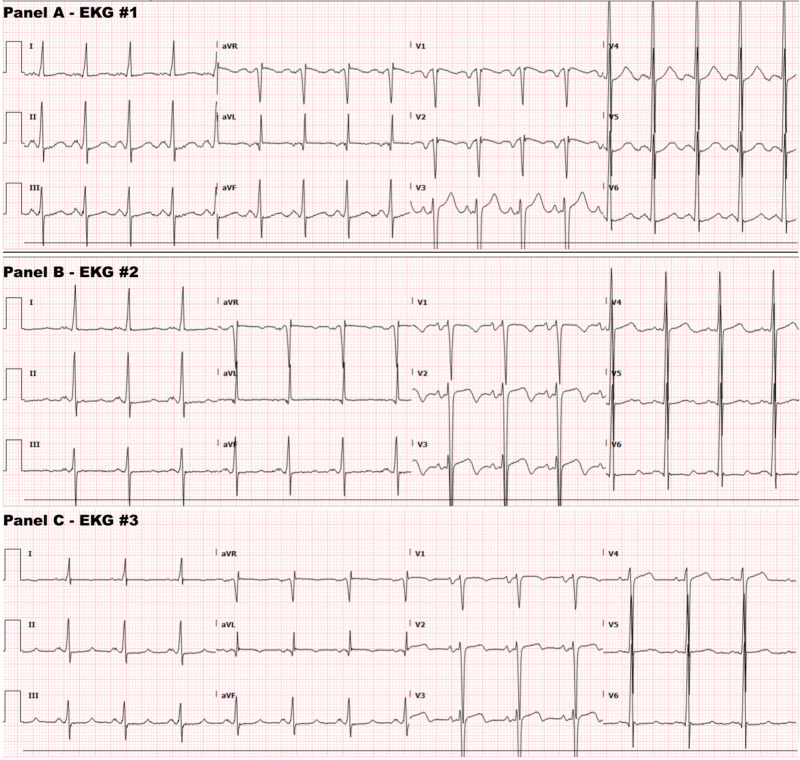
ECGs on admission ECG readings Panel A: HR 90 SR, indeterminate axis, T-wave inversion in V2 (no other ST-T changes), no pathological Q-waves, no p-mitrale or p-pulmonale Panel B: HR 100 SR, indeterminate axis, T-wave inversion in V2-V3 (no other ST-T changes), no pathological Q-waves, + p-mitrale in septal leads, no p-pulmonale Panel C: HR 85 SR, normal axis, T-wave inversion in aVL (no other ST-T changes), no pathological Q-waves, + p-mitrale in septal leads, no p-pulmonale Pathological Q-waves indicates signs of old infarctions. P-mitrale indicates left atrial enlargement. P-pulmonale indicates right atrial enlargement. Note: high-voltage criteria (QRS complexes) that may appear so in each of the above panels do not apply to patients less than 40 years of age. ECG, electrocardiogram.

**Figure 2:**
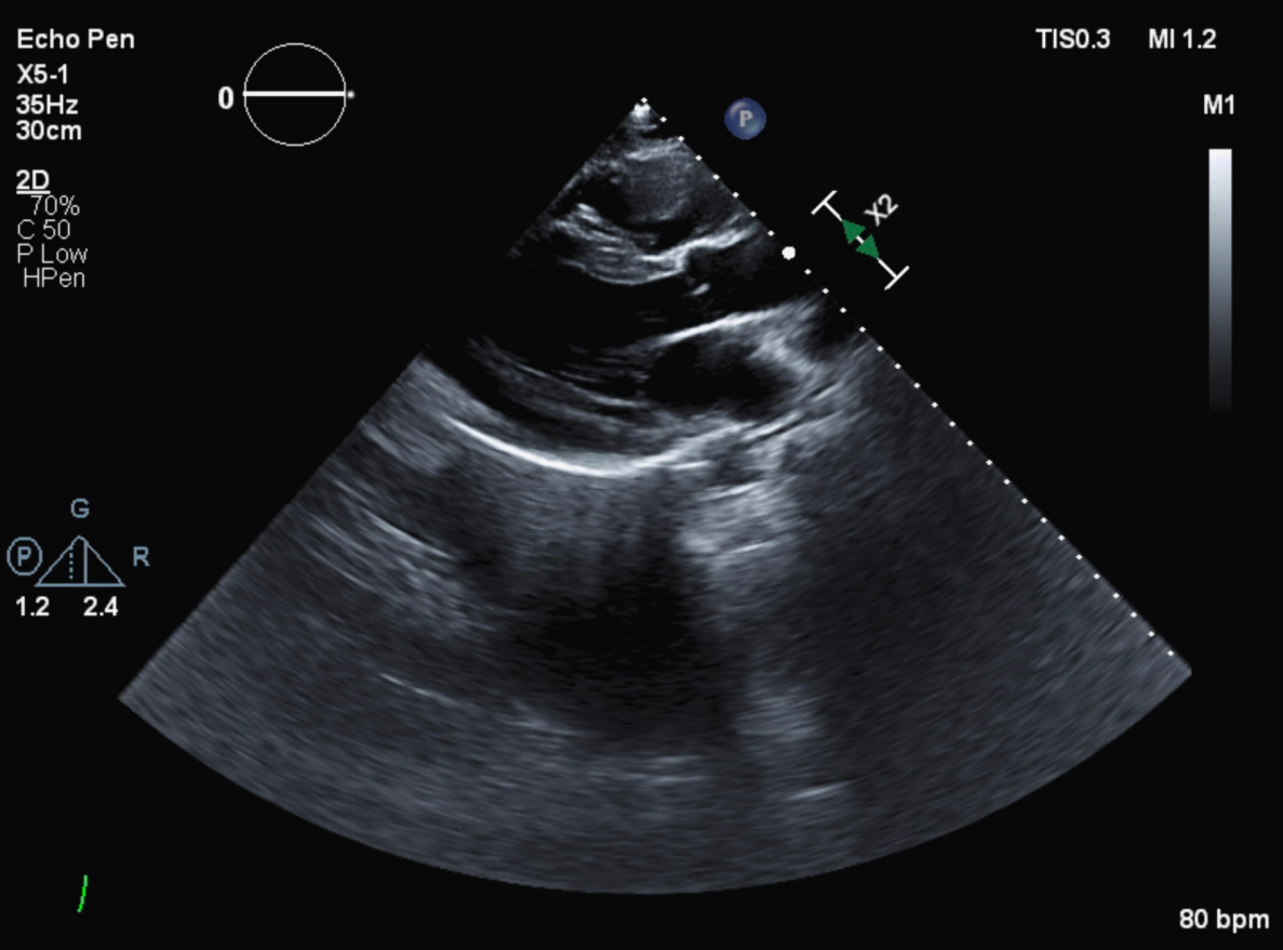
TTE Official report: Left ventricle: the chamber size is mildly enlarged. Wall thickness is mildly to moderately thickened. Systolic function is moderately reduced. The estimated ejection fraction is 40-45%. Moderate diffuse hypokinesis is present. Features are consistent with a pseudonormal left ventricular filling pattern, with concomitant abnormal relaxation and increased filling pressure (grade 2 diastolic dysfunction). Left atrium: the atrium is moderately dilated. Right ventricle: the chamber size is normal. Right atrium: the atrium is mildly dilated. Tricuspid valve: the valve appears structurally normal. Normal leaflet separation can be seen. Trivial regurgitation is noted. RVSP is 34. Pericardium: there is no pericardial effusion and no evidence of pleural fluid accumulation. TTE, transthoracic echocardiogram; RVSP, right ventricular systolic pressure.

Acute coronary syndrome was ruled out using several serial electrocardiograms (ECGs) (Figure 1). A transthoracic echocardiogram (TTE) yielded a left ventricular ejection fraction (LVEF) of 40-45% and mild-to-moderate left ventricular wall thickness, among other findings (Figure 2). Cardiac enzyme (troponin) levels were marginally elevated (Table 1). Left heart catheterization showed normal coronary arteries. Optimum medical therapy was instituted with a statin, beta-blocker, renin-angiotensin-aldosterone system (RAAS) antagonist, and arteriolar vasodilator. The aforementioned cardiac enzyme elevation was attributed to demand ischemia secondary to uncontrolled hypertension.

Given the presence of AKI on CKD stage II, a work-up of his non-nephrotic range proteinuria ensued. The nephrology service was consulted. A renal ultrasound showed an echogenic cortex bilaterally suggestive of chronic renal disease. A work-up for glomerulonephritis and secondary hypertension came up negative (Table 2). A subsequent renal artery angiogram was negative for any occlusion or vascular anomaly that could compromise renal blood flow. A renal biopsy revealed focal segmental glomerulosclerosis with microangiopathic changes, severe arteriolosclerosis, interstitial fibrosis, and atrophy. The diagnostic work-up of his uncontrolled hypertension itself was inconclusive given these findings.

**Table 2:** Glomerulonephritis and Secondary Hypertension work-up Note: values are normal unless otherwise indicated. Ig, immunoglobulin; L, low; HIV, human immunodeficiency virus; TPEP, total protein electrophoresis; SPEP, serum protein electrophoresis; H, high; ANA, anti-nuclear antibodies.

Test	Results
IgA	65 mg/dL (L)
IgG	702 mg/dL (L)
IgM	72 mg/dL
Acute hepatitis panel	Negative
HIV	Negative
TPEP	5.5 (L)
SPEP	Hypogammaglobulinemia with no monoclonal proteins
Albumin	3.34 g/dL (L)
Alpha-1 globulin	0.35 g/dL
Alpha-2 globulin	0.59 g/dL
Beta globulin	0.62 g/dL
Gamma globulin	0.60 g/dL (L)
Renin	16.9 μIU/mL (H)
Aldosterone	47.5 ng/dL (H)
ANA	Negative
C3	11 mg/dL (L)
C4	27 mg/dL
Glomerular basement membrane antibodies	Negative
Urine 24-hour protein	766 (H)

**Figure 3:**
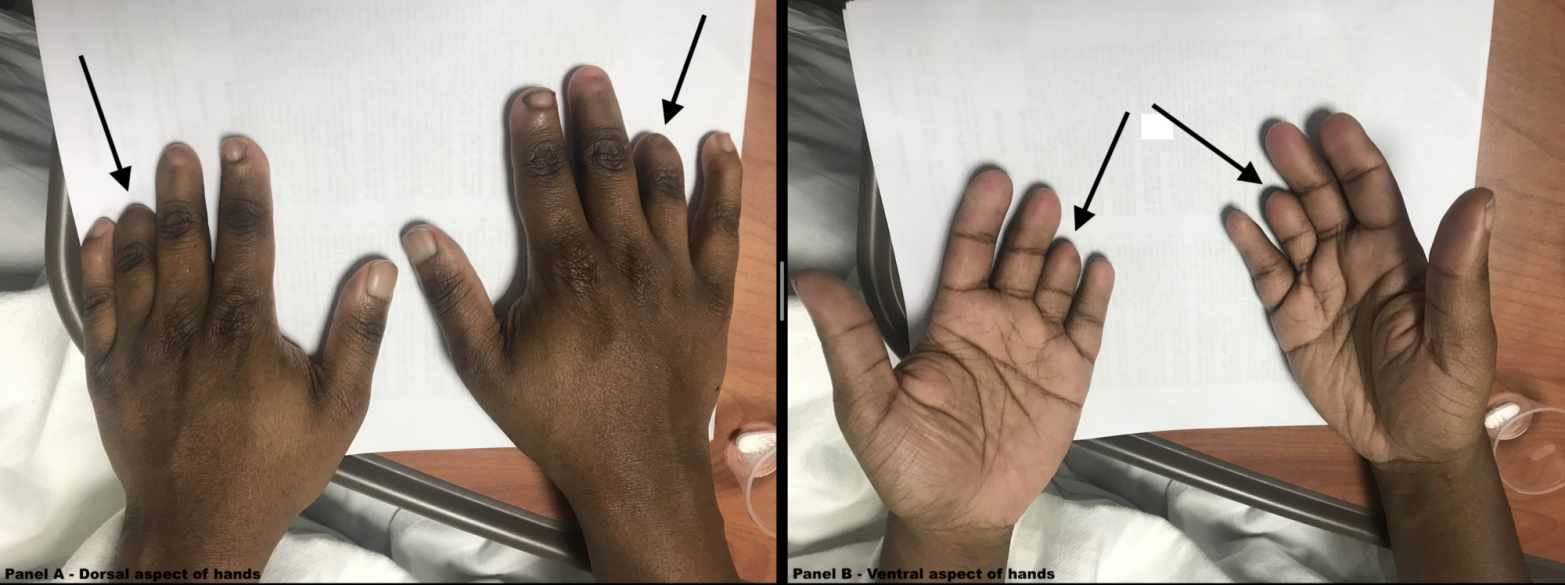
Photos of the hands Panel A: dorsal aspect of the hands. Panel B: ventral aspect of the hands. Arrows denoting prominent brachydactyly of the fourth digits of both hands.

**Figure 4:**
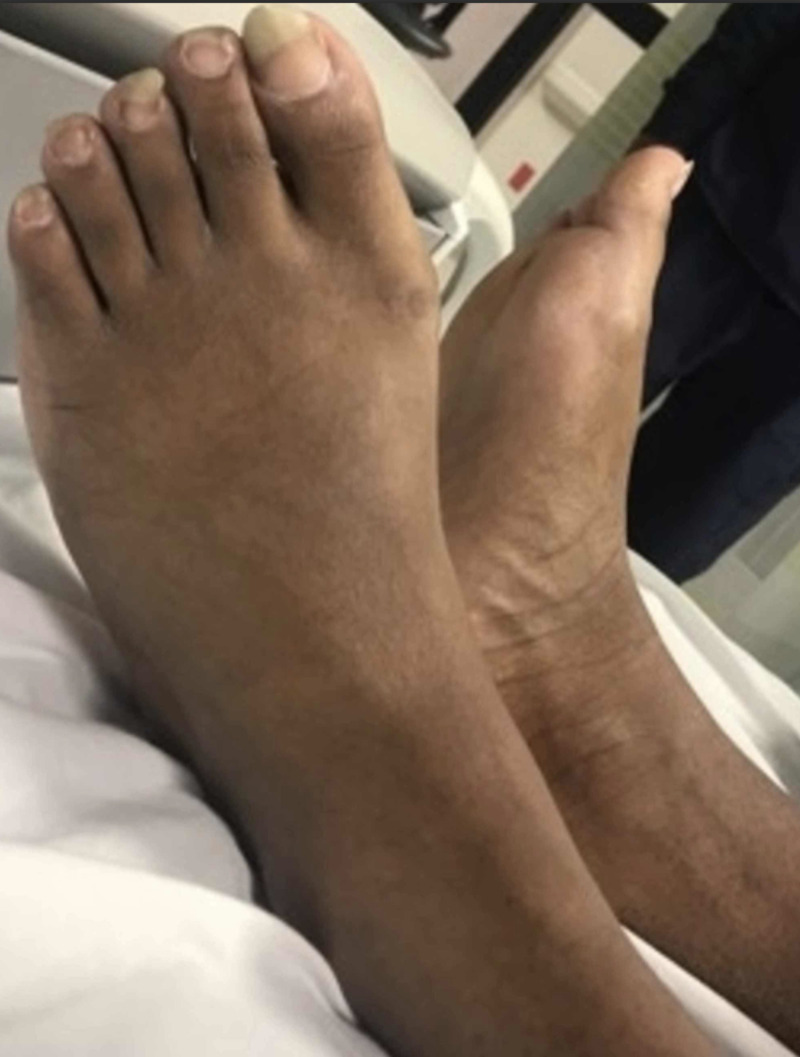
Dorsum of the feet (anatomically normal)

**Figure 5:**
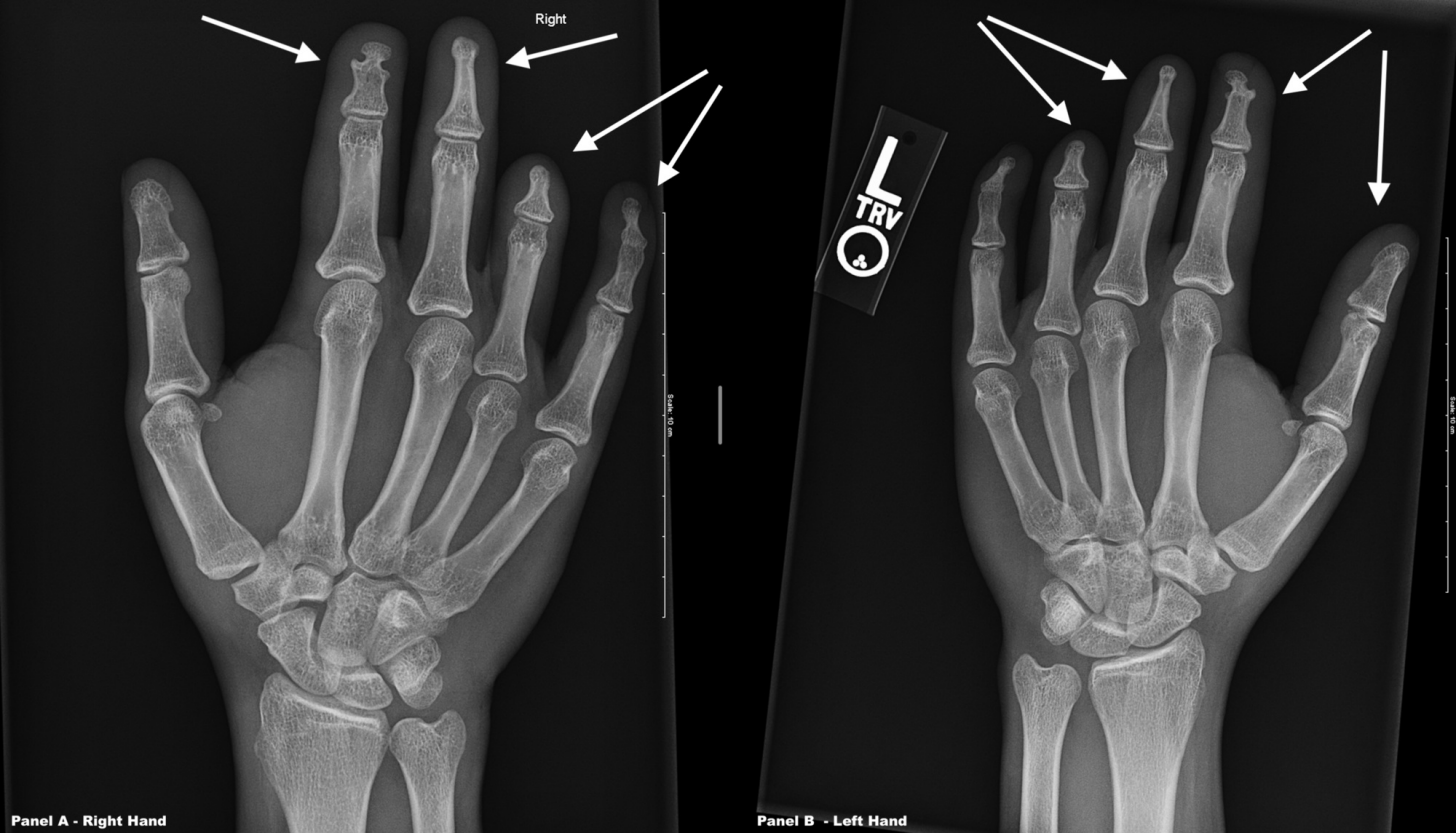
X-rays of both hands The arrows above denote the second through fifth digits. Radiology report: there is an abnormality at rays 2 through 5, with a similar appearance bilaterally. These include a truncated middle phalanx of the second ray, which may be partially fused with the distal phalanx. There is tapering and tubular configuration of the third ray middle phalanx and no visible distal phalanx. The fourth and fifth rays are equal length, and there is also tapering and truncation of the fourth ray middle phalanx and no visible distal phalanx. Mild clinodactyly of the fifth ray is seen, and there may be fusion at the fifth ray distal interphalangeal joint.

**Figure 6:**
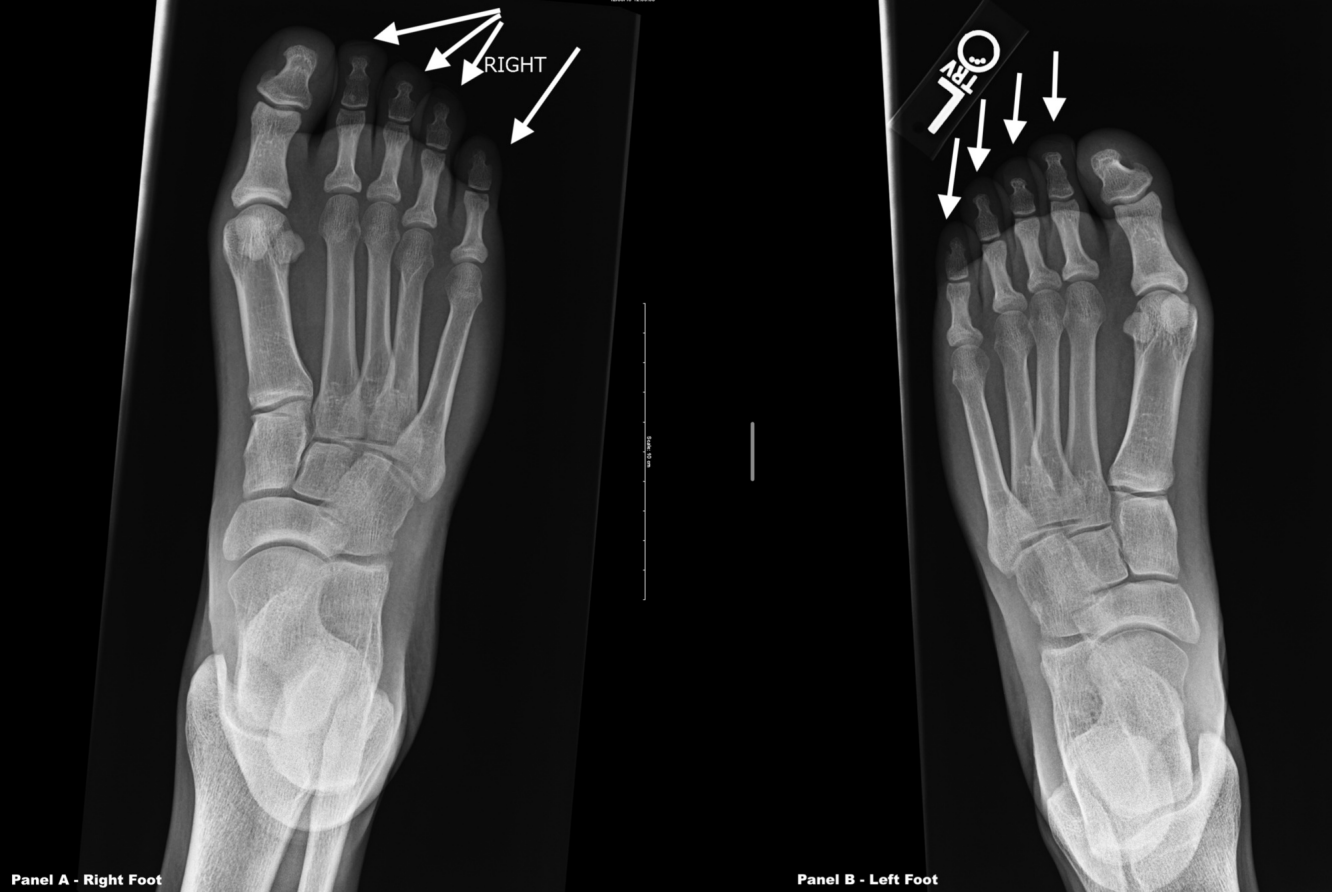
X-rays of both feet The arrows above denote the second through fifth digits. Radiology report: mild hallux valgus deformities are seen bilaterally. There is abnormal appearance to rays 2 through 5, with normal-appearing metatarsals and proximal phalanges. No distal phalanges are demonstrated at rays 2 through 5, and the middle phalanges appear slightly truncated with spade configuration. There may be a fusion of the middle phalanx and distal phalanx of the second toe, symmetric bilaterally. There is apparent absence or hypoplasia of the lateral cuneiform bones bilaterally.

Physical examination was impressive for the presence of brachydactyly of the bilateral hands, especially the fourth digits (Figure 3). His feet appeared grossly normal (Figure 4). X-rays (XRs) of the bilateral hands revealed absent distal phalanges in the third through fifth rays and fused middle and distal phalanges of the second digits (Figure 5). XRs of the bilateral feet showed absent distal phalanges of the second through fifth rays and spade configuration of the middle phalanx, in addition to the absence or hypoplasia of the lateral cuneiform bones bilaterally (Figure 6). As an aside, the patient did not have papilledema, and his pupils were equal and reactive to light and accommodation. He also had no appreciable murmur on cardiac examination. His neurologic examination was normal, including cranial nerves.

Given these findings in the setting of uncontrolled hypertension in a young adult, our patient was diagnosed with HTNB syndrome. The patient was discharged home. Genetic work-up could not be initiated since the patient failed to establish a follow-up visit in the clinic.

## Discussion

HTNB or Bilginturan syndrome is a rare autosomal dominant condition that was initially described by Bilginturan et al. in 1973 in a Turkish family [1]. Later cases have been found in Canadian and American families, both of English descent [2]. The disease itself is known to cause severe salt-independent, age-dependent hypertension beginning in childhood, which results in significant cerebrovascular disease before the age of 50 [2-3]. It is monogenic for which mutant gene is mapped to the short arm of chromosome 12. The resultant defective gene results in enhanced phosphorylation of the phosphodiesterase 3A (PDE3A) enzyme that results in accelerated vascular smooth muscle proliferation that causes peripheral vasoconstriction [3]. This sustained increase in systemic vascular resistance ultimately causes the uncontrolled hypertension described herein. Additionally, this defective enzyme results in dysregulated PTH-related protein (PTHrP) secretion yielding a pseudohypoparathyroidism-like state and subsequent brachydactyly and short stature [3-4]. Undiagnosed patients are very young and succumb to recurrent strokes. Unlike in our patient, hypertension-related end-organ damage is usually not apparent. An alternative pathophysiological mechanism for the uncontrolled hypertension component of this syndrome has been described. It has been suggested that a neurovascular anomaly in the posterior cranial fossa involving the posterior inferior cerebellar artery and vertebral arteries yields hypersensitivity of carotid and renal baroreceptors [5-6]. These anomalous vessels come directly into contact with the roots of cranial nerves IX and X, resulting in this heightened level of baroreceptor sensitivity [3,5-6]. CT and MRI studies did not show any such culprit vascular anomalies in our patient. Our patient’s uncontrolled hypertension was successfully managed with a beta-blocker, RAAS antagonist, and arteriolar vasodilator.

## Conclusions

HTNB or Bilginturan syndrome is a multiethnic disease. Clinicians should screen adolescents or young adults with secondary hypertension for brachydactyly. Early detection and treatment of HTNB will decrease the risk of detrimental end-organ damage and long-term debility in the form of recurrent cerebrovascular accidents such as strokes. Additionally, genetic testing should be considered in patients with suggestive HTNB phenotypes in order to facilitate family screening and pre-pregnancy genetic counseling.
